# A smartphone-supported weight loss program: design of the ENGAGED randomized controlled trial

**DOI:** 10.1186/1471-2458-12-1041

**Published:** 2012-11-30

**Authors:** Christine A Pellegrini, Jennifer M Duncan, Arlen C Moller, Joanna Buscemi, Alyson Sularz, Andrew DeMott, Alex Pictor, Sherry Pagoto, Juned Siddique, Bonnie Spring

**Affiliations:** 1 Current Address: Department of Preventive Medicine, Feinberg School of Medicine, Northwestern University, 680 N. Lake Shore Drive, Suite 1400, Chicago, IL, 60611, USA; 2 Department of Medicine, Division of Preventive and Behavioral Medicine, University of Massachusetts Medical School, 55 Lake Avenue North, Worcester, MA, 01655, USA

**Keywords:** Weight loss, Technology, Physical activity

## Abstract

**Background:**

Obesity remains a major public health challenge, demanding cost-effective and scalable weight management programs. Delivering key treatment components via mobile technology offers a potential way to reduce expensive in-person contact, thereby lowering the cost and burden of intensive weight loss programs. The ENGAGED study is a theory-guided, randomized controlled trial designed to examine the feasibility and efficacy of an abbreviated smartphone-supported weight loss program.

**Methods/design:**

Ninety-six obese adults (BMI 30–39.9 kg/m^2^) will be randomized to one of three treatment conditions: (1) standard behavioral weight loss (STND), (2) technology-supported behavioral weight loss (TECH); or (3) self-guided behavioral weight loss (SELF). All groups will aim to achieve a 7% weight loss goal by reducing calorie and fat intake and progressively increasing moderate intensity physical activity to 175 minutes/week. STND and TECH will attend 8 group sessions and receive regular coaching calls during the first 6 months of the intervention; SELF will receive the Group Lifestyle Balance Program DVD’s and will not receive coaching calls. During months 1–6, TECH will use a specially designed smartphone application to monitor dietary intake, body weight, and objectively measured physical activity (obtained from a Blue-tooth enabled accelerometer). STND and SELF will self-monitor on paper diaries. Linear mixed modeling will be used to examine group differences on weight loss at months 3, 6, and 12. Self-monitoring adherence and diet and activity goal attainment will be tested as mediators.

**Discussion:**

ENGAGED is an innovative weight loss intervention that integrates theory with emerging mobile technologies. We hypothesize that TECH, as compared to STND and SELF, will result in greater weight loss by virtue of improved behavioral adherence and goal achievement.

**Trial registration:**

NCT01051713

## Background

Obesity remains a pressing public health problem. Approximately 36% of adults in the United States are obese (Body Mass Index (BMI) ≥ 30 kg/m^2^) [1]. Globally, the World Health Organization estimates that approximately 500 million adults were obese in 2008 [2]. Obesity heightens the risk of a number of diseases, including type 2 diabetes, cardiovascular disease, obstructive sleep apnea, gallbladder disease, osteoarthritis, and several forms of cancer [3-6]. Consequently, obesity creates significant economic burden: U.S. medical costs associated with obesity were $147 billion in 2008 [7].

The Diabetes Prevention Program (DPP) intensive lifestyle intervention, which targets decreasing caloric and fat intake and increasing moderate-to-vigorous intensity physical activity, has been successful in producing clinically significant weight loss and reducing the incidence of diabetes in pre-diabetic adults [8]. In spite of its efficacy for weight loss and improving cardiometabolic outcomes, the DPP lifestyle intervention has not been widely adopted in its original form, primarily because it includes sixteen face-to-face sessions and sixteen to twenty-four optional maintenance meetings. This amount and type of contact is considered too burdensome and expensive to be a sustainable program [9]. In an attempt to increase the reach of this program, the DPP protocol has been translated for implementation in real-world settings [10] such as in a community YMCA [11] and a community-based diabetes education program led by community health workers [12]. Significant weight losses were produced ranging from 6.0-7.3% in both studies. However these programs may still have limited reach due to the 16–24 weekly group sessions required during months 1–6 and additional maintenance meetings during months 7–12 [11,12]. To date, studies that have reduced treatment intensity by reducing the number of in-person sessions have resulted in poorer weight loss outcomes [13]. Therefore, the still unmet challenge of DPP implementation is how to reduce in-person contact without eliminating features considered essential for successful weight loss: social support, accountability, and feedback.

### Technology and weight loss interventions

Technology-based interventions hold great promise as a mechanism for disseminating efficacious weight loss programs to a broader portion of the population via a cost-effective, scalable platform [14]. Many different technologies have been used in weight loss interventions including PDAs [15,16], cell phone text messages [17,18] and wearable physical activity monitors [19,20]. However, Internet-based interventions have received the most empirical attention to date [21]. Internet-based weight loss treatment findings have been mixed, and those programs that have achieved the highest weight losses have also included interactive tools, tailored feedback to participants, and in-person contact [21-24]. Programs that are solely internet-based however, do not allow for the same level of interactivity, social support, and feedback that occurs during in-person interventions.

New generation handheld technologies have the potential to overcome many of the limitations of internet-based health behavior change interventions [25]. Mobile phones are becoming ubiquitous: 46% of Americans own a smartphone, with rates even higher among minority groups [26]. Smartphones provide an unprecedented opportunity for frequent and interactive feedback, tailored email or text reminders, and immediate access to social support from peers and coaches. Interactive smartphone applications can be used as a decision support tool to provide timely information on health behavior decisions occurring in real-time. These technological advances afford a promising opportunity to preserve the key components of intensive in-person treatment approaches (i.e., regular social support, accountability, and frequent feedback) while requiring fewer in-person sessions, thus, lowering the cost and broadening the accessibility of treatment. To test the feasibility and efficacy of this mobile technology-supported strategy, we have developed the E-Networks Guiding Adherence to Goals in Exercise and Diet (ENGAGED) study, a randomized controlled trial (RCT) that uses a theory-guided, technology-supported weight loss program.

### Theoretical framework

The conceptual basis for the ENGAGED study is Carver and Scheier’s control systems theory (CST) of self-regulation [27]. CST posits that self-regulation can be understood in terms of feedback loops, wherein people: (1) set a goal, (2) self-monitor their current status to identify discrepancy from a goal, and then (3) modify their behavior to reduce perceived discrepancy. From a CST perspective, DPP’s efficacy can be attributed to helping participants (1) set appropriate caloric intake and physical activity goals, (2) self-monitor weight, eating behavior, and activity, and (3) adjust behaviors to reach the targets. According to CST, the core challenge in weight loss is aligning diet and activity with goals. Most weight loss efforts lose because traditional paper and pencil self-monitoring of diet and activity fails to provide salient, timely decision support and accurate feedback.

Using CST as a guiding framework, ENGAGED will develop a persuasive, interactive smartphone application designed improve the saliency, timeliness, and accuracy with which participants receive feedback on their daily dietary and activity goals. Participants will monitor dietary intake and wear a Bluetooth-enabled accelerometer to receive real-time feedback on objectively measured physical activity. Persuasively designed goal thermometers on the application will display current behavior and goals to highlight behavior-goal discrepancies. We posit based on CST that the experience of receiving consistent and immediate feedback from the ENGAGED technology should reinforce adherence to self-monitoring and healthy behavior change, both during and after in-person treatment ends.

### Social networks, peer support, and weight loss

Research on social networks and weight has elicited promising results. Christakis and Fowler [28] reported that obesity can spread virally through social networks. Relatedly, other findings suggest that including friends and family as co-participants in weight loss treatment improves outcomes [29]. However, not all overweight and obese adults have family or friends willing to join and/or support them in their weight loss efforts. Thus, additional strategies to provide peer support need to be identified. Social networking tools have gained popularity on many online commercial weight management programs, and preliminary evidence indicates that technology-supported social networking can be beneficial. Krukowski et al. [30] found that use of social networking features in an online weight management program was positively associated with weight loss maintenance.

The ENGAGED study will utilize technology that enhances social networking capabilities on a smartphone application to promote the development and maintenance of a positive social network. The application will allow teammates who have been incentivized to help each other via a group weight loss competition to view each others’ adherence to daily self-monitoring goals. Additionally, the application allows team members to communicate with each other via private message board or peer-to-peer messaging. By capitalizing on team-based performance incentives and enabling team building through the ENGAGED social networking technology, we aim to extend support, encouragement, and accountability for behavioral change, between and after in-person group sessions.

### Study aims

Guided by CST and social networks research, the ENGAGED study will evaluate the feasibility and efficacy of a technology-supported weight loss program for obese adults. Specifically, the primary aim of the ENGAGED study is to conduct a three-group randomized controlled trial testing the efficacy of the ENGAGED technology-supported behavioral weight loss program, as compared to the same program delivered using standard paper and pencil self-monitoring, and self-guided behavioral weight loss supported by participants receiving DVD’s depicting DPP treatment. Primary outcomes include: (1) weight loss and (2) behavioral adherence, which is operationalized by (a) self-monitoring of diet and activity and (b) attainment of diet and activity goals. As a secondary outcome, the time spent administering the intervention by lifestyle coaches will also be examined to assess intervention costs. The second aim of the ENGAGED study is to test whether the weight loss in the technology-supported condition is mediated by behavioral adherence to self-monitoring and achievement of diet and activity goals.

We hypothesize that using a theory-guided, technology-supported weight loss treatment will result in clinically significant weight loss with fewer in-person sessions by preserving regular social support, accountability, and feedback via mobile technology. Further, we hypothesize that technology-supported weight loss treatment will result in greater weight loss compared to the traditional paper self-monitoring approach.

## Methods

### Study design

The research design is a 3 × 4 factorial with one between subjects factor (treatment condition) with three levels and one within subjects factor (time) with four levels (baseline, 3-, 6-, and 12-months). Participants will be randomized to one of three groups: a) Standard behavioral weight loss program (STND); b) technology-supported behavioral weight loss program (TECH); or c) self-guided behavioral weight loss program (SELF).

### Eligibility

A total of 96 participants who meet the inclusion and exclusion criteria listed in Table 1 will be recruited in two cohorts. Participants will be between 18–60 years old with a body mass index (BMI) between 30–40 kg/m^2^, weight stable for the past 6 months, not currently enrolled in a formal weight loss program or taking weight loss medications, and be willing to self-monitor and wear an accelerometer daily for 6 months. Participants will be excluded if they have any unstable medical conditions, are currently pregnant, or taking any medications known to influence weight. See Table 1 for a complete list of exclusion criteria.

**Table 1:** ENGAGED inclusion and exclusion criteria

**Inclusion**	**criteria**
	• BMI 30.0-39.9 kg/m^2^
	• 18-60 years old
	• Interested in losing weight through diet and physical activity changes
	• Weight stable over the past 6 months
	• Not receiving concurrent weight loss treatment
	• Willing to self-monitor (on smartphone or paper) and wear an accelerometer daily for 6 months
	• Able to attend all assessments and group sessions
	• Have or be willing to learn basic technology usage skills
**Exclusion**	**criteria**
	• Has one or more of the following unstable medical conditions: uncontrolled hypertension or diabetes, angina pectoris, myocardial infarction, plantar fasciitis, transient ischemic attack, cancer undergoing active treatment, or cerebrovascular accident within the past 6 months
	• Has insulin-dependent diabetes, Crohn’s Disease, obstructive sleep apnea requiring use of a CPAP, plantar fasciitis
	• Requires use of an assistive device for mobility
	• Has been hospitalized for a psychiatric disorder in the past 5 years
	• Contraindications for moderate intensity physical activity
	• Currently taking any weight-loss medications or committed to following an incompatible dietary regimen
	• Currently pregnant, trying to get pregnant, or lactating
	• Current bulimia nervosa or binge eating disorder
	• Current substance or alcohol or dependence
	• Current attention deficit hyperactivity disorder or major depressive disorder
	• Currently taking medication(s) known to cause weight gain (i.e. Prednisone, Depakote, Diebeta, Diabinese, Cardura, Inderal, Zyprexa)
	• Expresses active suicidal ideation

### Recruitment and screening process

**Figure 1:**
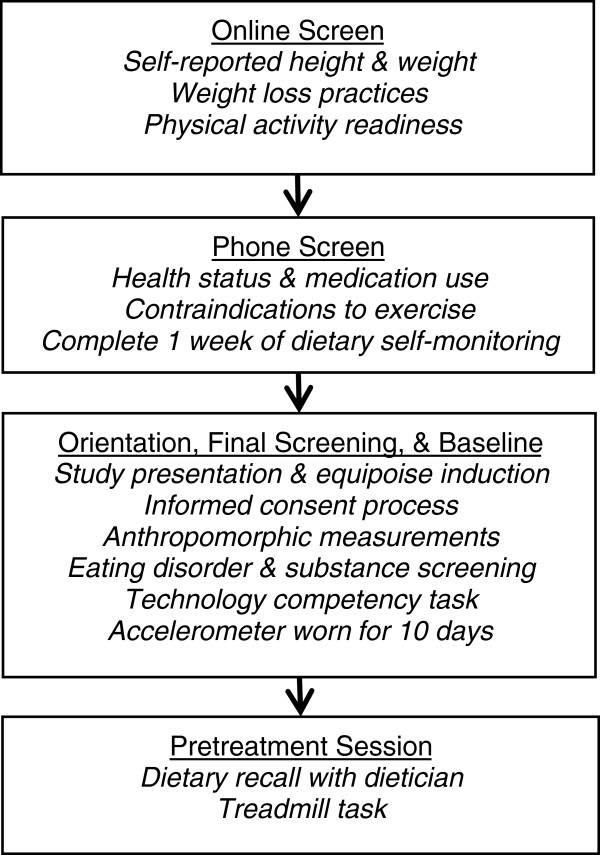
ENGAGED Study Screening Flowchart.

All study procedures are approved by the Institutional Review Board at Northwestern University. Participants will be recruited via flyers and advertisements in local newspapers and on public transportation. Interested candidates will be directed to a study website which includes study-related information and an online screening questionnaire to assess initial eligibility. Figure 1 illustrates the screening process.

#### Online screening

Participants will complete an initial online consent process prior to completing the online screener, which assesses height and weight, physical activity readiness, group session availability, and exclusionary weight loss practices (i.e. participation in a weight loss program or use of weight loss medications). Study staff will contact eligible participants within 1–4 days of completing an online screener to conduct a telephone screen.

#### Telephone screening

The telephone screen will review details of the study and review additional eligibility criteria including health status and medication use. Eligible and interested participants will be invited to attend a study orientation session and will be instructed to begin a one-week run-in period of dietary self-monitoring. If participants indicate a health condition such as type II diabetes or hypertension, medical clearance from a physician will be required prior to the pretreatment session in order to continue participation.

#### In-person orientation session

During the orientation session, complete details of the study will be given and an equipoise induction will be performed to ensure that participants are aware of the pros and cons of the treatment conditions and to minimize the risk of differential attrition [31]. Participant questions will be answered and informed consent will be obtained from interested participants. Consented participants will complete the final in-person screening and baseline assessment, which includes assessing blood pressure and anthropometric measurements. In addition, participants will complete a smartphone competency task to evaluate their ability to use basic functions of a smartphone after instruction and will complete a brief interview to assess: binge eating disorder, bulimia nervosa, substance abuse and dependence, attention deficit hyperactivity disorder, and major depressive disorder. Eligible participants will also complete baseline questionnaires and wear an accelerometer for 10 days to measure current activity levels.

#### Pre-treatment session

At the pretreatment session, participants will return the accelerometer, meet with a dietician to complete a dietary recall, and engage in a brief treadmill activity. During the treadmill activity, participants will walk on a treadmill at a moderate intensity while reviewing ratings of perceived exertion to become familiar with the physiological signs of physical activities at this intensity.

### Randomization

**Figure 2:**
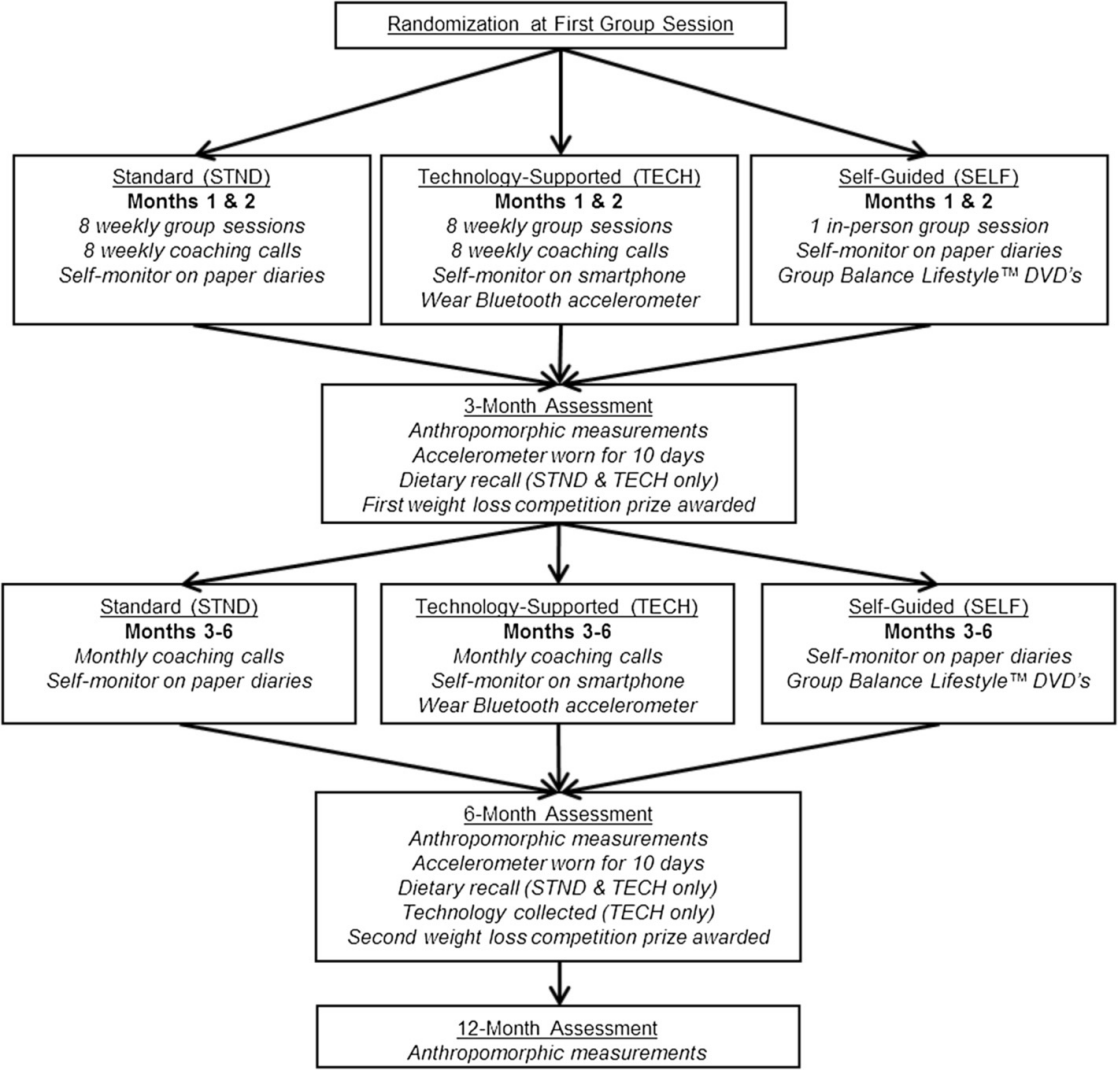
ENGAGED Intervention Timeline.

Eligible participants will be scheduled for a group time and assigned to a team with seven other participants, all of whom will be randomized to the same condition. Group randomization will be completed by a statistician, and neither participants, nor coaches, will find out group assignment until the first group session. The intervention timeline is presented in Figure 2.

### Standard weight loss program (STND)

STND participants will be given a goal of 7% weight loss to be achieved through changes in diet and activity, similar to the DPP lifestyle intervention [32]. Based on baseline weight, participants will be given daily calorie goal between 1200–2000 kcal/day and a 25% fat gram goal between 32–55 grams/day. These goals are designed to produce a one to two pound weight loss per week. Participants will also be given a weekly physical activity goal, beginning with 45 minutes of moderate intensity physical activity per week and progressing weekly to 175 minutes per week. Participants will be provided with “The Complete Book of Food Counts” [33] and paper Keeping Track diaries during months 1–6 to facilitate self-monitoring of dietary intake, physical activity, and body weight.

The original sixteen session DPP protocol will be condensed to eight group sessions (see Table 2) and, similar to the Look AHEAD trial [34], group sessions will be combined with individual telephone coaching to increase social support and reduce costs [35]. Group sessions will run 90 minutes in duration and be held weekly for the first eight weeks of the study. Each group session will begin with a private weigh-in and participants will submit their Keeping Track diary. All group sessions will be led by a trained lifestyle interventionist and will include setting an agenda, reviewing homework and self-monitoring, presentation of new material, group discussions, and assignment of homework. See Table 2 for a list of session topics. All group sessions will end with an optional supervised group walking session.

**Table 2:** ENGAGED Study Group Session Topics

**Session**	**Topics covered**
1	Getting Started With Activity and Healthy Eating
	Be a Fat Detective
2	Three Ways to Eat Less Fat
	Four Keys to Healthy Eating Out
3	Take Charge of What’s Around You: Making Cues Work for You
4	Move Those Muscles and Be Active As A Way of Life!
5	You Can Manage Stress
	Talk Back to Negative Thoughts
6	Tip the Calorie Balance
	Problem Solving
7	Healthy Eating
	The Slippery Slope of Lifestyle Change
8	Jump Start Your Activity Plan
	Ways to Stay Motivated

In addition to in-person group sessions, STND participants will receive individual coaching calls. Coaching calls will be scheduled on a weekly basis during the initial eight weeks of the study and monthly during months 3–6. Coaches will review submitted Keeping Track diaries and determine the participant’s level of achievement for each goal using standardized adherence criteria for the study. Coaching calls will last approximately 10–15 minutes and follow a structured script aiming to reinforce success and problem solve around barriers to change. Four content areas will be covered during each call: (1) weight loss, (2) adherence to dietary and physical activity self-monitoring, (3) attainment of diet and activity goals, and (4) goal setting.

### Technology-supported weight loss program (TECH)

Similar to STND, TECH will attend eight in-person group sessions, receive regular coaching calls, and follow the same weight, physical activity, and dietary goals. However rather than paper Keeping Track diaries, TECH will be provided with a study smartphone, ENGAGED application, and an accelerometer to use during the first 6 months of the intervention. In addition to covering the topics discussed during the first group meeting (Table 2), TECH will be instructed on how to self-monitor dietary intake, use the accelerometer, and communicate virtually with their teammates and coach using the ENGAGED application. Time-stamped dietary and physical activity data will be continuously downloaded to a secure server, and participant data will be summarized automatically on the secure coaching application and website, allowing coaches to have up-to-date access to participant data, enabling corrective or supportive feedback to be given via text message or during scheduled coaching calls.

### ENGAGED technology

**Figure 3:**
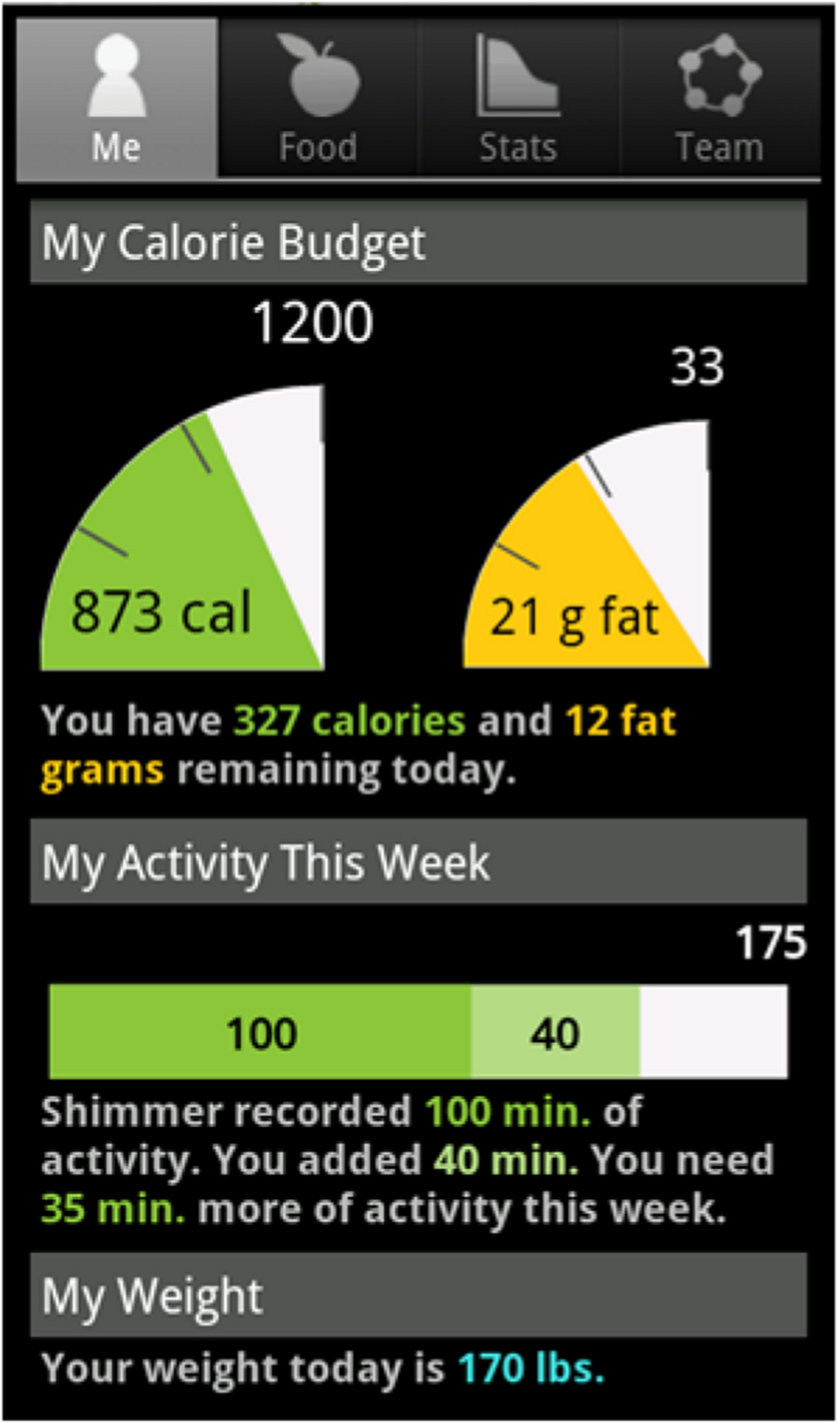
Screen shot of ENGAGED smartphone application.

The ENGAGED technology includes a study smartphone (Motorola Droid™), pre-loaded with the ENGAGED application, and an accelerometer (SHIMMER™). TECH participants will use the ENGAGED application, which contains the CalorieKing® food database, a comprehensive nutritional source containing over 50,000 food entries, to self-monitor daily dietary intake. Goal thermometers will display participant’s goal and actual amount of calories and fat grams consumed (Figure 3). The ENGAGED application also includes a Team tab that allows participants to view team members’ adherence to self-monitoring and accelerometer usage. In addition, the Team tab on the application includes a message board and peer-to-peer messaging to facilitate communication and support among teammates. Participants will also wear an accelerometer that connects via Bluetooth to the smartphone to provide real-time objective data on the accumulation of moderate-to-vigorous intensity physical activity. In the event participants engage in an activity not captured by the accelerometer (i.e. cycling, swimming), users may manually input physical activity.

### Self-guided behavioral weight loss program (SELF)

SELF will attend one 60-minute in-person group session during but will not attend any additional group sessions nor receive coaching calls throughout the intervention. Similar to STND, SELF will be given “The Complete Book of Food Counts” [33] and paper Keeping Track diaries. Rather than attend in-person group sessions, SELF will receive three Group Lifestyle Balance™ (GLB) Program DVDs [36] that contain 12 group sessions adapted from the original DPP curriculum as well as the corresponding GLB participant handouts. SELF will be given their individual 7% weight loss goals and instructed to watch the DVDs to get their calorie and fat gram goals.

### Team weight loss competition

In order to encourage members of each weight loss team to support each other’s behavioral adherence and weight loss, a team weight loss competition will be held at 3- and 6-months during each cohort. The team with the greatest weight loss percentage at each time point will win $400, to be divided evenly among the group members ($50 per team member).

### Treatment fidelity

Fidelity checklists will be prepared for group and telephone coaching sessions. Checklists for each session will specify a) good counseling practices; b) prescribed session content; and c) proscribed session content. Coaches will undergo training to become familiarized with the modified DPP protocol and to role-play potential conflicts and situations that may arise during group and telephone sessions. Study staff will also attend weekly clinical meetings to discuss any participant conflicts that may arise. All group and telephone coaching sessions will be audio recorded, and each month a random sample of 10% of the recordings will be coded by study staff to evaluate treatment fidelity. If fidelity falls below 90% on any of the fidelity checks, staff will be retrained.

### Outcome measures

Assessments will be completed at baseline, 3-, 6-, and 12-months. Participants will receive $40 for completing each of the 3-, 6, and 12-month assessments. The procedures and measures are described below:

#### Height, weight, BMI, and waist circumference

Height will be measured using a wall-mounted stadiometer to the nearest 0.01 cm. Body weight will be measured without shoes, wearing light clothing on a calibrated beam balance scale to the nearest 0.01 kg. BMI (kg/m^2^) will be calculated using height and weight measurements. Waist circumference will be assessed by positioning an anthropometric tape midway between the palpated iliac crest and the palpated lowest rib margin in the mid-axillary lines. Two measurements will be taken and the mean value of each site will be used.

#### Dietary intake adherence

Dietary intake will be assessed at baseline, 3-, and 6-months via interview by a non-intervention registered dietician using the computer-assisted telephone interview (CATI) 24 hour recalls [37] using the multiple pass method to assess calorie and fat intakes. At each time point, three days (one weekend and two weekdays) will be randomly selected over a three week period to conduct the recalls. The data derived from the recalls will be analyzed using the annually updated University of Minnesota Nutrition Coordinating Center’s (NCC) Nutrition Data System for Research (NDS-R-2005). Only STND and TECH will complete dietary recalls at 3- and 6-months.

#### Physical activity adherence

Physical activity will be objectively measured by an accelerometer (Actigraph #7164, Pensacola, FL) over 7 days at baseline, 3-, and 6-months. The accelerometer will be attached to an elastic belt and worn on the right hip. Minutes of moderate-to-vigorous intensity physical activity will be measured in a modified bout lasting a minimum of eight consecutive minutes. Adherence will be ascertained on the basis of how well the participant’s bout-corrected accelerometer count corresponds to the weekly activity goal.

#### Dietary self-monitoring adherence

Dietary self-monitoring adherence will be assessed as the number of days reporting dietary intake on the paper Keeping Track diaries (STND and SELF) or smartphone application (TECH). In order to receive full adherence credit for a day of self-monitoring, participants must have ≥ 1000 calories recorded and ≥ 25 grams of fat.

#### Physical activity self-monitoring adherence

For STND and SELF, physical activity self-monitoring adherence will be assessed as the number of days reporting physical activity in the paper Keeping Track diaries, divided by the number of possible days. For TECH participants, self-monitoring adherence will be operationalized as the number of days the accelerometer was worn ≥ 8 hours, divided by the number of possible days.

#### Coaching time

Coaches will monitor the number of minutes preparing for and conducting individual coaching calls throughout the first 6 months of the intervention.

### Data analytic plan

#### Linear mixed modeling

Data will be analyzed on an intent-to-treat basis. The basic modeling approach will entail linear mixed models for longitudinal data. The primary aim is to test for treatment group differences on weight loss at 3-, 6-, and 12-months as well as differences in behavioral adherence and goal attainment at 3- and 6-months. Linear mixed models do not require that participants are measured at all time points, and therefore can include participants with missing data across time. In these analyses, we will examine changes across time by treating time as a nominal variable in the analysis to allow for non-linear trends. In addition to the effect of time, the main independent variable is treatment groups, a between-subjects factor with 3 levels: STND, TECH and SELF. A priori Helmert contrasts [38] will be used to test the following comparisons at 3- and 6-months: 1) STND and TECH vs. SELF and 2) TECH vs. STND. We will also examine group by time interactions to test whether change in outcome across time differs by group.

### Mediation analysis

We hypothesize that the weight loss advantage for those assigned to TECH would be explained (entirely or partially) by TECH’s higher rates of adherence to self-monitoring and attainment of behavioral goals. To test this mediation hypothesis, we will fit a linear mixed model predicting weight loss similar to the model described above, but now including in the regression equation the potential mediating variables corresponding to adherence to self-monitoring. To the extent that adherence mediate the effects of the experimental condition, in the model with adherence included, we expect the coefficient on the treatment group by time interaction variable to be reduced compared to the treatment group by time interaction coefficient from the model without adherence. This difference provides an estimate of the extent to which adherence may mediate the effects of the experimental condition on weight loss. Bootstrapping will be used to estimate the sampling variability of the mediation effect.

### Sample size and power

We will randomize n=32 participants to each treatment group and assume 6% of participants will drop out by the end of the study so that there will be n=30 participants in each group at the final endpoint. The power calculations below are based on the second Helmert contrast: TECH vs. STND because this contrast contains the fewest number of subjects (30 in each group) and we expect this contrast to have the smallest effect sizes. The first Helmert contrast: STND and TECH vs. SELF has a larger sample size (60 vs. 30) and we anticipate larger effects sizes in this contrast due to the limited intensity of the SELF intervention. Therefore, by powering the study based on the second contrast, we will have more than adequate power for the first contrast.

#### Weight loss

We measure weight loss at 3, 6, and 12 months. Using data from a previous weight loss trial [16], we saw standard deviations of 8.4 and 10.7 pounds at months 3 and 6, respectively, with a correlation of 0.86 between the two time points. With 80% power and an n=30 in both groups, we will be able to detect a difference of 6.6 pounds between the two groups.

#### Diet self-monitoring adherence

We measure dietary self-monitoring adherence at 3 and 6 months as the ratio of actual days of diet self-monitoring relative to the possible number of days. Using data from a previous trial [16], we observed a standard deviation of 0.31 and 0.34 at 3 and 6 months, respectively, as well as a correlation of 0.93 between the two time points. Thus, with 80% power and an n=30 in both groups, we will be able to detect a difference in adherence ratios of 0.23 between the two groups.

#### Physical activity self-monitoring adherence

Physical activity self-monitoring will be assessed as the number of days reporting physical activity on paper diaries (STND and SELF) or wearing the accelerometer (TECH), divided by the number of possible days. Data from a previous trial [16] shows a standard deviation of 0.30 at 3 months and 0.33 at 6 months and a correlation of 0.91 between the two time points. With 80% power and an n=30 in both groups, we will be able to detect a difference in percent adherence of 22% between the two groups.

#### Dietary intake adherence

We measure adherence to calorie goals at 3 and 6 months using 3 day 24 hour recalls. Using pilot data from a previous trial, we observed a standard deviation of change in calories of 498 at month 3 and 431 at month 6, with a correlation of 0.9 between the two time points. With 80% power and an n=30 in both groups, we will be able to detect a calorie reduction difference of 375 calories.

#### Physical activity adherence

Physical activity adherence is measured by accelerometer at months 3 and 6 as the number of minutes of moderate to vigorous intensity physical activity per week. Using data from a previous trial [39], we assume a standard deviation of 142 minutes/week at months 3 and 6, with a correlation of 0.9 between the two time points. With 80% power and an n=30 in both groups, we will be able to detect a 100 minute/week difference between the two groups.

## Discussion

Obesity continues to be a significant public health issue worldwide and guidelines recommend intensive behavioral interventions for all obese adults [1,2,40]. However, efficacious and scalable interventions have not yet been widely implemented. Mobile technology provides an opportunity to develop interventions with lower costs, less burden, and a greater reach, particularly as mobile usage rates continue to rise. As of February 2012, approximately 46% of American adults currently own a smartphone, an increase from 36% in May 2011 [26]. Minority groups, including Blacks and Hispanics saw an increase in smartphone use from 44% to 49% during this same time period [26]. Smartphones have the ability to extend key components of intensive weight loss treatment into real-world settings, while reducing treatment costs and maintaining efficacious weight loss outcomes. The ENGAGED study is an innovative randomized controlled trial that will examine the feasibility and efficacy of a theory-guided, technology-supported weight loss program. Specifically, a total of 96 obese adults will be randomized to one of three conditions: a) Standard behavioral weight loss program (STND); b) technology-supported behavioral weight loss program (TECH); or c) self-guided behavioral weight loss program (SELF). Differences in weight loss, behavioral adherence, and goal attainment will be examined between groups.

The technology-supported condition will utilize a smartphone application designed to target known health behavior change mechanisms. Participants will be able to track weight and dietary intake, receive real-time feedback on behaviors, and communicate with team members by using a private message board and direct messaging. The application will also connect wirelessly via Blue-tooth to an accelerometer to provide real-time feedback on objectively measured physical activity. The ENGAGED technology will enable participants to experience ongoing support and accountability by allowing study coaches to continuously view participant’s up-to-date data, enabling them to provide timely support in the event of technical difficulties and tailored, immediate feedback during coaching calls.

Limited research has been conducted on smartphone-supported weight loss interventions, leaving numerous unanswered questions and directions for future research. Future studies are especially needed on the efficacy of mobile health interventions in underrepresented groups (i.e., older adults, ethnic minorities, uneducated, and low SES). Also needed are studies on the efficacy of technology supported interventions over the long-term, as weight loss maintenance continues to be one of the greatest challenges to obesity interventions [41]. To date, there are few or no behavioral interventions that fully maintain weight losses after treatment ends [42]. If the ENGAGED study provides preliminary evidence that a smartphone-supported intervention improves short-term weight loss and adherence outcomes, further studies will be warranted to examine whether the use of this technology can enhance long-term weight loss maintenance.

The ENGAGED Study is a theory-guided randomized controlled trial that will examine the efficacy and feasibility of a smartphone-supported implementation of the DPP on weight loss and behavioral adherence. Although smartphone usage is on the rise, the evidence base evaluating applications for health behavior change is extremely sparse. This study will make an important contribution by rigorously examining the effect of a novel, scalable smartphone technology system on self-monitoring adherence and weight loss outcomes.

## Abbreviations

CST: Control systems theory; DPP: Diabetes prevention program; SELF: Self-guided behavioral weight loss; STND: Standard behavioral weight loss; TECH: Technology-supported behavioral weight loss.

## Competing interests

The authors declare no competing interests.

## Authors’ contributions

BS, SP, AM, and JS were responsible for study conceptualization, design, and developing the analytic plan. CP, JD, JB, AS, AD, and AP participated in operationalizing the intervention. CP, JD, AM, JB, and BS drafted the manuscript. All authors read and approved the final manuscript.

## Pre-publication history

The pre-publication history for this paper can be accessed here:

http://www.biomedcentral.com/1471-2458/12/1041/prepub

## References

[B1] Flegal KM Carroll MD Kit BK Ogden CL Prevalence of obesity and trends in the distribution of body mass index among US adults, 1999–2010 JAMA 2012 307 5 491 497 10.1001/jama.2012.39 22253363

[B2] WHO Obesityhttp://www.who.int/topics/obesity/en/

[B3] Resnick HE Valsania P Halter JB Lin X Relation of weight gain and weight loss on subsequent diabetes risk in overweight adults J Epidemiol Community Health 2000 54 8 596 602 10.1136/jech.54.8.596 10890871 PMC1731720

[B4] Mokdad AH Marks JS Stroup DF Gerberding JL Actual causes of death in the United States, 2000 JAMA 2004 291 10 1238 1245 10.1001/jama.291.10.1238 15010446

[B5] Must A Spadano J Coakley EH Field AE Colditz G Dietz WH The disease burden associated with overweight and obesity JAMA 1999 282 16 1523 1529 10.1001/jama.282.16.1523 10546691

[B6] Bianchini F Kaaks R Vainio H Overweight, obesity, and cancer risk Lancet Oncol 2002 3 9 565 574 10.1016/S1470-2045(02)00849-5 12217794

[B7] Finkelstein EA Trogdon JG Cohen JW Dietz W Annual medical spending attributable to obesity: payer- and service-specific estimates Health Aff 2009 28 5 W822 W831 10.1377/hlthaff.28.5.w822 19635784

[B8] Knowler WC Barrett-Connor E Fowler SE Hamman RF Lachin JM Walker EA Nathan DM Reduction in the incidence of type 2 diabetes with lifestyle intervention or metformin N Engl J Med 2002 346 6 393 403 11832527 10.1056/NEJMoa012512 PMC1370926

[B9] Eddy DM Schlessinger L Kahn R Clinical outcomes and cost-effectiveness of strategies for managing people at high risk for diabetes Ann Intern Med 2005 143 4 251 264 16103469 10.7326/0003-4819-143-4-200508160-00006

[B10] Whittemore R Melkus G Wagner J Dziura J Northrup V Grey M Translating the diabetes prevention program to primary care: a pilot study Nurs Res 2009 58 1 2 12 10.1097/NNR.0b013e31818fcef3 19092550 PMC2689783

[B11] Ackermann RT Finch EA Brizendine E Zhou HH Marrero DG Translating the diabetes prevention program into the community - the DEPLOY pilot study Am J Prev Med 2008 35 4 357 363 10.1016/j.amepre.2008.06.035 18779029 PMC2610485

[B12] Katula JA Vitolins MZ Rosenberger EL Blackwell CS Morgan TM Lawlor MS Goff DC Jr One-year results of a community-based translation of the diabetes prevention program: healthy-living partnerships to prevent diabetes (HELP PD) project Diabetes Care 2011 34 7 1451 1457 10.2337/dc10-2115 21593290 PMC3120203

[B13] Cramer JS Sibley RF Bartlett DP Kahn LS Loffredo L An adaptation of the diabetes prevention program for use with high-risk, minority patients with type 2 diabetes Diabetes Educ 2007 33 3 503 508 10.1177/0145721707301680 17570881

[B14] Coons MJ DeMott A Buscemi J Duncan JM Pellegrini CA Steglitz J Pictor A Spring B Technology interventions to curb obesity: a systematic review of the current literature Curr Cardiovasc Risk Rep 2012 6 120 134 10.1007/s12170-012-0222-8 23082235 PMC3471367

[B15] Burke LE Styn MA Sereika SM Conroy MB Ye L Glanz K Sevick MA Ewing LJ Using mHealth technology to enhance self-monitoring for weight loss: a randomized trial Am J Prev Med 2012 43 1 20 26 10.1016/j.amepre.2012.03.016 22704741 PMC3563396

[B16] Spring B Duncan JM Janke EA Kozak AT McFadden HG Demott A Pictor A Epstein LH Siddique J Pellegrini CA Integrating technology into standard weight loss treatment: a randomized controlled trial Arch Intern Medin press 10.1001/jamainternmed.2013.1221 PMC3684245 23229890

[B17] Patrick K Raab F Adams MA Dillon L Zabinski M Rock CL Griswold WG Norman GJ A text message-based intervention for weight loss: randomized controlled trial J Med Internet Res 2009 11 1 e1 10.2196/jmir.1100 19141433 PMC2729073

[B18] Haapala I Barengo NC Biggs S Surakka L Manninen P Weight loss by mobile phone: a 1-year effectiveness study Public Health Nutr 2009 12 12 2382 2391 10.1017/S1368980009005230 19323865

[B19] Pellegrini CA Verba SD Otto AD Helsel DL Davis KK Jakicic JM The comparison of a technology-based system and an in-person behavioral weight loss intervention Obesity (Silver Spring) 2012 20 2 356 363 10.1038/oby.2011.13 21311506 PMC3753800

[B20] Shuger SL Barry VW Sui X McClain A Hand GA Wilcox S Meriwether RA Hardin JW Blair SN Electronic feedback in a diet- and physical activity-based lifestyle intervention for weight loss: a randomized controlled trial Int J Behav Nutr Phys Act 2011 8 41 10.1186/1479-5868-8-41 21592351 PMC3112373

[B21] Neve M Morgan PJ Jones PR Collins CE Effectiveness of web-based interventions in achieving weight loss and weight loss maintenance in overweight and obese adults: a systematic review with meta-analysis Obes Rev 2010 11 4 306 321 10.1111/j.1467-789X.2009.00646.x 19754633

[B22] Saperstein SL Atkinson NL Gold RS The impact of Internet use for weight loss Obes Rev 2007 8 5 459 465 10.1111/j.1467-789X.2007.00374.x 17716303

[B23] Tate DF Jackvony EH Wing RR Effects of Internet behavioral counseling on weight loss in adults at risk for type 2 diabetes - A randomized trial JAMA 2003 289 14 1833 1836 10.1001/jama.289.14.1833 12684363

[B24] Tate DF Jackvony EH Wing RR A randomized trial comparing human e-mail counseling, computer-automated tailored counseling, and no counseling in an Internet weight loss program Arch Intern Med 2006 166 15 1620 1625 10.1001/archinte.166.15.1620 16908795

[B25] Patrick K Griswold WG Raab F Intille SS Health and the mobile phone Am J Prev Med 2008 35 2 177 181 10.1016/j.amepre.2008.05.001 18550322 PMC2527290

[B26] Pew internet & American life project: Smartphone update 2012 http://pewinternet.org/Reports/2012/Smartphone-Update-2012.aspx

[B27] Carver C Scheier M On the self regulation of behavior 1998 New York: Cambridge University Press

[B28] Christakis NA Fowler JH The spread of obesity in a large social network over 32 years N Engl J Med 2007 357 4 370 379 10.1056/NEJMsa066082 17652652

[B29] Wing RR Jeffery RW Benefits of recruiting participants with friends and increasing social support for weight loss and maintenance J Consult Clin Psychol 1999 67 1 132 138 10028217 10.1037//0022-006x.67.1.132

[B30] Krukowski RA Harvey-Berino J Ashikaga T Thomas CS Micco N Internet-based weight control: the relationship between web features and weight loss Telemed J e Health 2008 14 8 775 782 10.1089/tmj.2007.0132 18954247 PMC2998280

[B31] Goldberg JH Kiernan M Innovative techniques to address retention in a behavioral weight-loss trial Health Educ Res 2005 20 4 439 447 10.1093/her/cyg139 15598664

[B32] Program TDP Design and methods for a clinical trial in the prevention of type 2 diabetes Diabetes Care 1999 22 4 623 634 10189543 10.2337/diacare.22.4.623 PMC1351026

[B33] Netzer CT The complete book of food counts 2006 7th New York: Dell Pub

[B34] Ryan DH Espeland MA Foster GD Haffner SM Hubbard VS Johnson KC Kahn SE Knowler WC Yanovski SZ Look AHEAD (Action for Health in Diabetes): design and methods for a clinical trial of weight loss for the prevention of cardiovascular disease in type 2 diabetes Control Clin Trials 2003 24 5 610 628 10.1016/S0197-2456(03)00064-3 14500058

[B35] Renjilian DA Perri MG Nezu AM McKelvey WF Shermer RL Anton SD Individual versus group therapy for obesity: effects of matching participants to their treatment preferences J Consult Clin Psychol 2001 69 4 717 721 11550739

[B36] Kramer MK Kriska AM Venditti EM Miller RG Brooks MM Burke LE Siminerio LM Solano FX Orchard TJ Translating the diabetes prevention program: a comprehensive model for prevention training and program delivery Am J Prev Med 2009 37 6 505 511 10.1016/j.amepre.2009.07.020 19944916

[B37] Posner BM Borman CL Morgan JL Borden WS Ohls JC The validity of a telephone-administered 24-hour dietary recall methodology Am J Clin Nutr 1982 36 3 546 553 6180624 10.1093/ajcn/36.3.546

[B38] Bock RD Multivariate statistical methods in behavioral research 1975 New York: Mc-Graw Hill

[B39] Spring B Schneider K McFadden HG Vaughn J Kozak AT Smith M Moller AC Epstein LH Demott A Hedeker D Multiple behavior changes in diet and activity: a randomized controlled trial using mobile technologybehavior changes in diet and Activity Arch Intern Med 2012 172 10 789 796 10.1001/archinternmed.2012.1044 22636824 PMC3402206

[B40] USPSTFecommendations - Obesity in adultshttp://www.uspreventiveservicestaskforce.org/recommendations.htm

[B41] Elfhag K Rossner S Who succeeds in maintaining weight loss? A conceptual review of factors associated with weight loss maintenance and weight regain Obes Rev 2005 6 1 67 85 10.1111/j.1467-789X.2005.00170.x 15655039

[B42] Svetkey LP Stevens VJ Brantley PJ Appel LJ Hollis JF Loria CM Vollmer WM Gullion CM Funk K Smith P Comparison of strategies for sustaining weight loss: the weight loss maintenance randomized controlled trial JAMA 2008 299 10 1139 1148 10.1001/jama.299.10.1139 18334689

